# AEG‐1 induces gastric cancer metastasis by upregulation of eIF4E expression

**DOI:** 10.1111/jcmm.13258

**Published:** 2017-06-29

**Authors:** Shengjie Wu, Li Yang, Dandan Wu, Zhongyuan Gao, Ping Li, Wenbin Huang, Xuerong Wang

**Affiliations:** ^1^ Department of Pharmacology Nanjing Medical University Nanjing Jiangsu China; ^2^ Department of General Surgery Medical Oncology and Pathology the First Affiliated Hospital of Nanjing Medical University Nanjing Jiangsu China; ^3^ Department of Basic Medicine Kangda College of Nanjing Medical University Lianyungang Jiangsu China; ^4^ Department of Pathology Nanjing Medical University Affiliated Nanjing Hospital (Nanjing First Hospital) Nanjing Jiangsu China

**Keywords:** gastric cancer, metastasis, epithelial–mesenchymal transition, AEG‐1, eIF4E

## Abstract

Gastric cancer is the third leading cause of cancer‐related deaths worldwide, and patients with lymph node, peritoneal and distant metastasis have a poor prognosis. Overexpression of Astrocyte‐elevated gene‐1 (AEG‐1) has been reported to be correlated with the progression and metastasis of gastric cancer. However, its mechanisms are quite unclear. In this study, we found that elevated expression of AEG‐1 was correlated with metastasis in human gastric cancer tissues. Moreover, gain‐ or loss‐of‐function of AEG‐1, respectively, promoted or suppressed epithelial–mesenchymal transition (EMT), migration and invasion of gastric cancer cells. AEG‐1 positively regulated eIF4E, MMP‐9 and Twist expression. Manipulating eIF4E expression by transfection of overexpression constructs or siRNAs partially eliminated AEG‐1‐regulated EMT, cell migration and invasion. In addition, overexpression or knockdown of eIF4E promoted or suppressed EMT, cell migration and invasion in parallel with upregulation of MMP‐9 and Twist expression, while manipulating eIF4E expression partially abrogated AEG‐1‐induced MMP‐9 and Twist. Finally, silencing of AEG‐1 expression not only inhibited tumour growth in parallel with downregulation of eIF4E, MMP‐9 and Twist expression in a xenograft nude mouse model, but also suppressed lymph node and peritoneal metastasis of gastric cancer in an orthotopic nude mouse model. These findings suggest that AEG‐1 promotes gastric cancer metastasis through upregulation of eIF4E‐mediated MMP‐9 and Twist, which provides new diagnostic markers and therapeutic targets for cancer metastasis.

## Introduction

Gastric cancer is the fifth most common malignancy and the third leading cause of cancer‐related deaths worldwide 1. Although the incidence and mortality rates of gastric cancer have decreased in recent years due to early diagnosis and considerable advances in surgical, chemo‐, radio‐ and other adjuvant therapies, especially the treatment of *Helicobacter pylori* infections, the 5‐year survival rate is still only 30% 2. Most patients are diagnosed at a late stage with lymph node or distant metastases, or with relapse after prior curative surgical therapy 3. Moreover, peritoneal metastasis is the most frequent metastatic and recurrent site, which has a very poor prognosis 4. The identification of molecules that play important roles in metastasis and clarification of their mechanisms will provide novel diagnostic markers and therapeutic targets for gastric cancer.

AEG‐1, also known as metadherin (MTDH) or lysine‐rich CEACAM‐1 associated protein (LYRIC), was first identified and characterized in primary human astrocytes infected with HIV 5, 6, 7. AEG‐1/MTDH was subsequently found to mediate the metastasis of breast cancer 8. In recent years, AEG‐1 expression was found to be elevated and correlated with clinical tumour type, stage, metastasis and prognosis in a variety of cancers, including breast 9, lung 10, colorectal 11, cervical 12, 13, head and neck 14, hepatocellular 15 and gastric cancers 16, 17. AEG‐1 has been shown to be transcriptionally up‐regulated by Ha‐Ras‐activated PI3K and c‐Myc 18. Moreover, AEG‐1 activates the PI3K/Akt, MAPK/ERK, NF‐κB and Wnt signalling pathways to promote cell proliferation, survival, metabolism, EMT, migration, invasion, protective autophagy and angiogenesis 19, 20. However, the biological roles of AEG‐1 in the metastasis of gastric cancer and its mechanisms have not been elucidated.

The eukaryotic translation initiation factor 4E (eIF4E) is a component of the translational initiation complex eIF4F 21. It binds to the mRNA 5′ cap structure and facilitates recruitment of mRNA to ribosomes for the subsequent translation of mRNA 22, 23. Previous research proved that eIF4E plays a pivotal role in cell growth, survival, invasion, EMT and angiogenesis, and that it promotes tumorigenesis, metastasis, and recurrence in numerous cell and animal models 24. Overexpression of eIF4E was reported in many types of cancers, suggesting it as a diagnostic marker and therapeutic target 25. EIF4E activity is regulated by the PI3K/Akt/mTOR/4E‐BP1, MEK/ERK/Mnk and p38 MAP kinase pathways 26, 27. Mnk1/2 phosphorylation activates eIF4E directly. C‐Myc is the only factor that regulates eIF4E expression transcriptionally 28. EIF4E specifically regulates the expression of some cancer‐related mRNAs, including c‐Myc, cyclin D1, Bcl‐2, Mcl‐1, MMP‐2, MMP‐9 and VEGF 29.

We previously reported that AEG‐1 is up‐regulated in gastric dysplasia and cancer, and its high expression is correlated with the Lauren classification (*P* = 0.027), the T classification (*P* = 0.001), the N classification (*P* = 0.002) and pTNM staging (*P* = 0.043) 16. We also found that eIF4E expression was increased in gastric tumour tissue compared with adjacent non‐cancerous tissue, and its overexpression was correlated with distant metastasis 30. Moreover, both of these important molecules are targets of perifosine, a synthetic alkylphosphocholine that inhibits Akt signalling 16, 30. As AEG‐1 is reported to be located downstream of PI3K/Akt and to function as a transcriptional factor, we suspected that eIF4E may be transcriptionally regulated by AEG‐1. In this study, we first evaluated the correlation between AEG‐1 overexpression and metastasis of gastric cancer in human cancer specimens, then determined the role of AEG‐1 in EMT, migration, and invasion of gastric cancer cells in parallel with the detection of eIF4E expression, *via* overexpression or silencing of AEG‐1. We also examined the effects of eIF4E overexpression or knockdown on AEG‐1‐regulated EMT, cell migration and invasion, and investigated EMT regulators, such as MMP‐9 and Twist, that mediate eIF4E's function. In addition, we examined the effect of AEG‐1 silencing on gastric cancer metastasis in an orthotopic transplantation nude mouse model.

## Materials and methods

### Reagents

Lipofectamine 2000^®^ transfection reagent (11668‐019) was purchased from Life Technologies Co. Invitrogen (Carlsbad, CA, USA). AEG‐1/MTDH antibodies (13860‐1‐AP) were purchased from Zhongshan Goldenbridge Biotechnology Co., Ltd. (Beijing, China). EIF4E antibody (9742) was purchased from Cell Signaling Technology, Inc. (Beverly, MA, USA). E‐cadherin (BS1098), N‐cadherin (BS2224), MMP‐9 (BS1241) and actin (AP0060) antibodies were purchased from Bioworld Technology, Inc. (Louis Park, MN, USA). Twist antibody (ab175430) was purchased from Abcam (Cambridge, UK).

### Cell lines and cell culture

Two human gastric cancer cell lines, MGC803 and SGC7901, were obtained from the Shanghai Institutes for Biological Sciences, Chinese Academy of Sciences, China. Lentivirus containing short hairpin RNA (shRNA) of AEG‐1 (AEG‐1_shRNA) or control (scramble_shRNA) was purchased from Genechem (Shanghai, China). The target sequence of the AEG‐1 shRNA was 5′‐GCAGCAAGGCAGUCUUUAAGU‐3′, which was proved in our previous study 16. The AEG‐1 stable knockdown and the control cell lines were established by infection of the MGC803 (MGC803‐AEG‐1_shRNA/MGC803‐scramble_shRNA) and SGC7901 (SGC7901‐AEG‐1_shRNA/SGC7901‐scramble_shRNA) cells with the lentivirus for 48 hrs. They were then selected with puromycin (5 mg/ml) for 14 days and then withdrawn from puromycin for another 14 days as previously described 31. Cells were cultured in RPMI‐1640 medium (Hyclone, Logan, UT, USA) supplemented with 10% foetal bovine serum (FBS, Gibco BRL, Grand Island, NY, USA) at 37°C in a humidified atmosphere of 5% CO_2。._

### Quantitative real‐time polymerase chain reaction (qRT‐PCR)

Total RNA from cells was extracted using TRIzol^®^ reagent (1596‐026) from Invitrogen Life Technologies (Carlsbad, CA, USA), and reverse transcription was conducted using RevertAid™ Reverse Transcriptase (EP0441) from Thermo Fisher Scientific, Inc. (Rockford, IL, USA). Quantitative PCR was conducted using FastStart Universal SYBR Green PCR Master mix (4913914001) (Rox; 11929100) from Roche (Indianapolis, IN, USA), according to the manufacturer's instructions. The forward (F) and reverse (R) primers were synthesized by Invitrogen as follows: AEG‐1, F: 5′‐ACGACCTGGCCTTGCTGAAGAATCT‐3′ and R: 5′‐CGGTTGTAAGTTGCTCGGTGGTAA‐3′; eIF4E, F: 5′‐CCTACAGAACAGATGGGCACTC‐3′ and R: 5′‐GCCCAAAAGTCTTCAACAGTATCA‐3′; GAPDH, F, 5′‐ATGGGGAAGGTGAAGGTCG‐3′ and R, 5′‐GGGGTCATTGATGGCAACAATA‐3′ 32, 33. All measurements were performed in triplicate and measured with the ABI Prism 7300 sequence detection system (Applied Biosystems, Foster City, CA, USA), as previously described. The fold‐change of AEG‐1 and eIF4E was calculated using the 2^−ΔΔCT^ method.

### Western blot analysis

Whole‐cell protein lysates were prepared and subjected to Western blotting as described previously 16.

### Gene silencing by small interfering RNA (siRNA)

Control (non‐target) siRNA and eIF4E siRNA pools that target 5′‐GGACGAUGGCUAAUUACAU‐3′ 34; 5′‐AAGCAAACCUGCGGCUGAUCUTT‐3′ (our design) were used as previously described and synthesized by GenePharma Co., Ltd. (Shanghai, China). Cells were seeded in 6‐well plates at 5 × 10^5^ cells/well, transfected with 100 nmol/l siRNA using Lipofectamine 2000 and then subjected to subsequent performances.

### Plasmid construction and cell treatment

The AEG‐1 overexpression plasmid, a kind gift from Dr. Kunmei Liu (Ningxia Medical University), was constructed by insertion of full‐length AEG‐1 into the pcDNA vector as previously described 35. The eIF4E overexpression plasmid, a kind gift from Dr. Shi‐Yong Sun from Emory University, was constructed by inserting the coding sequence of eIF4E into the p3 × flag‐CMV‐14 plasmid as previously described 36.

### Transwell migration assay and Matrigel^®^ invasion assay

Cell migration and invasion were evaluated using a 24‐well Millicell^®^ hanging cell culture inserts (8.0 μm pore size; PIEP12R48) purchased from Millipore Corporation (Billerica, MA, USA) with or without Matrigel (B&D Biosciences, San Jose, CA, USA). Cells (5 × 10^4^) starved overnight were added to the upper chamber in serum‐free medium, and the bottom chamber was filled with conditioned medium containing 10% FBS. After 24 hrs of incubation, the cells in the upper chamber were wiped and removed with a cotton swab. Cells that migrated or invaded to the other side of the chamber were fixed with 100% ethanol and then stained for 30 min. with 0.1% crystal violet. The cells were then counted under a microscope at 100× magnification. Five fields from each sample were randomly selected, and the average number of cells in each field was counted and presented as mean ± S.D.

### Xenograft and orthotopic nude mouse models

Animal experiments were approved by the Institutional Animal Care and Use Committee of Nanjing Medical University. Athymic nude mice (BALB/C‐nu/nu, 4–5 weeks old, female) were purchased from Beijing Vital River Laboratory Animal Technology Co., Ltd. (Beijing, China), and 1 × 10^7^ SGC7901‐AEG‐1_shRNA cells and the control SGC7901‐scramble_shRNA cells were inoculated subcutaneously into their flank regions (*n* = 6 mice per group). The length and width of the tumours were measured every 3 days, and tumour volume (mm^3^) was calculated as π/6 (width^2^ × length). When the experiment was complete, the mice were killed and the tumours were removed, weighed and chilled in liquid nitrogen for storage at −80°C. Tissue protein lysates were prepared and subjected to Western blot assays.

When a xenograft reached 5 mm, the tumour was isolated and sheared into 1–2 mm^3^ pieces. The tumour fragments were again inoculated subcutaneously into the mouse's flank region. This transplantation procedure was repeated for five generations. Tumour tissues from the fifth generation were minced and transplanted into the seromuscular layer of the greater curvature of the stomach (*n* = 10mice per group). One or two drops of medical anastomotic glue OB (Guangzhou Baiyun Medical Adhesive Co., Ltd., Guangzhou, China) was used to seal the serosal layer incision, which was then covered with the greater omentum. When the mouse's abdomen became swollen (~1.0 cm × 1.0 cm), we removed the orthotopically transplanted tumour and calculated the number of metastatic nodules. This orthotopic model was previously described by Hu *et al*. 37. Tissue samples were fixed in 10% formalin and embedded in paraffin. Sections of 5 μm thickness were prepared for haematoxylin and eosin (H&E) staining, which was performed in a semi‐automated dyeing machine (Sakura, Japan) according to the manufacture's specifications. Finally, the sections were mounted on the mounting machine (Leica, Germany).

### Human tissue samples and immunohistochemical assay

Specimens from 87 cases of gastric adenocarcinoma were stained with AEG‐1 antibody as previously reported 16. We re‐read the immunochemistry staining of AEG‐1 and analysed the correlations between AEG‐1 expression and lymph node metastasis using chi‐square test. *P* < 0.05 was determined as statistically significant.

### Statistical analysis

The statistical significance of differences between different treatments was analysed with the two‐sided unpaired Student's *t*‐test. Results were considered statistically significant at *P* < 0.05.

## Results

### Elevated expression of AEG‐1 correlated with gastric cancer metastasis

We have previously reported that AEG‐1 is up‐regulated in gastric dysplasia and cancer, but we did not analyse its relationship with cancer metastasis 16. Therefore, we re‐read the histological sections of 87 cases of gastric adenocarcinoma with AEG‐1 immunohistochemistry staining and conducted a statistical analysis of the metastases. As shown in Table 1, stronger positive staining of AEG‐1 was observed in tumours with lymph node metastasis than in tumours without metastasis. The chi‐square test showed statistical significance, suggesting that increased expression of AEG‐1 is correlated with gastric cancer metastasis.

**Table 1:** Correlation between AEG‐1 expression and lymph node metastasis

Lymph node status	*n*	AEG‐1 expression		χ^2^	*P* value
		High	Low		
Metastasis					
Yes	54	41	13	13.466	0.000
No	33	12	21		

### Knockdown of AEG‐1 expression inhibited EMT, migration and invasion of gastric cancer cells in parallel with downregulation of eIF4E expression

**Figure 1:**
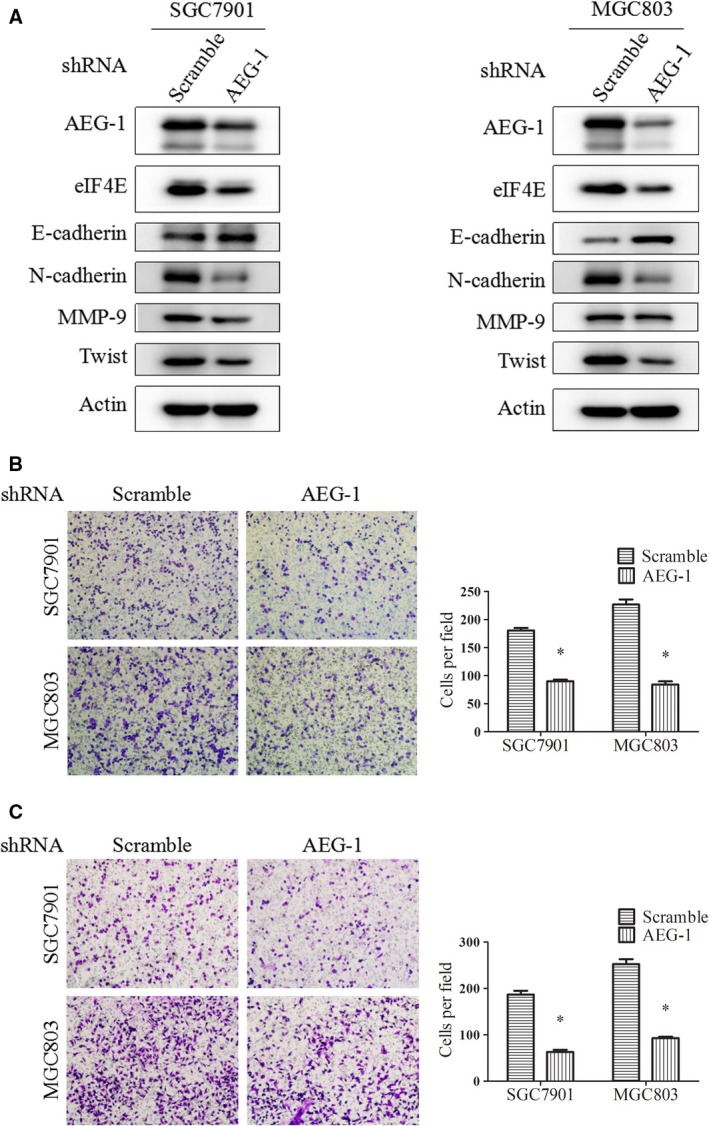
Knockdown of AEG‐1 expression suppressed EMT, migration and invasion of gastric cancer cells. **A**, SGC7901 and MGC803 cells with stable AEG‐1 knockdown (AEG‐1_shRNA) or control cells (scramble_shRNA) were cultured for 24 hrs and then subjected to Western blot assay. **B** and **C**, transwell migration assay (**B**) and Matrigel invasion assay (**C**) of AEG‐1 knockdown cells. Cell lines were seeded into the upper chamber in serum‐free medium. The lower chamber was filled with conditioned medium containing 10% FBS. After 24 hrs, cells in the lower chamber were subjected to the assay and observed under microscopy. The average number of cells in the lower chamber was quantified. Magnification, 100×. Columns, means of five fields; bars, S.D. *P < 0.05. The data are representatives of three independent experiments.

We previously reported that transient knockdown of AEG‐1 expression by siRNA inhibited the growth of gastric cancer cells 16. To further explore the role of AEG‐1 in metastastatic cells, we established two pairs of AEG‐1 stable knockdown cell lines in SGC7901 and MGC803 cells, as they had elevated expression of AEG‐1 and are commonly used for migration and invasion studies 16, 30. The SGC7901 and MGC803 cells were infected with lentivirus carrying AEG‐1 shRNA or control shRNA and then selected with puromycin for 2 weeks. As shown in Data S1, the mRNA and protein levels of AEG‐1 were significantly decreased in SGC7901‐AEG‐1_shRNA and MGC803‐AEG‐1_shRNA cells compared to the control cell lines, SGC7901‐scramble_shRNA and MGC803‐scramble_shRNA, suggesting successful knockdown of AEG‐1. We then detected the expression of EMT markers in these cell lines. Western blot assay showed that the epithelial marker (E‐cadherin) increased and the mesenchymal marker (N‐cadherin) decreased in the AEG‐1‐knockdown cells compared with the control cells, suggesting reversal of EMT (Fig. 1A). Furthermore, the transwell migration assay showed that cell numbers in the lower chambers were decreased in the AEG‐1‐silencing cells compared with the control cells, suggesting inhibition of cell migration (Fig. 1B). Statistical significance was observed after cell‐number quantification. In addition, the Matrigel invasion assay showed that AEG‐1 knockdown significantly decreased the invasive capacity of the cells (Fig. 1C). These results suggested that downregulation of AEG‐1 expression inhibited EMT, cell migration and invasion.

To examine the regulation of eIF4E by AEG‐1, we detected eIF4E expression in these AEG‐1‐knockdown cells in parallel. As shown in Figure 1A, eIF4E expression was significantly decreased in the AEG‐1‐knockdown cells compared with the control cells. As eIF4E has been reported to play an important role in EMT and cancer metastasis, we suspected that it might be a downstream target of AEG‐1 and may mediate the oncogenic effect of AEG‐1.

### Overexpression of AEG‐1 induced EMT, migration and invasion of gastric cancer cells, as well as upregulation of eIF4E expression

**Figure 2:**
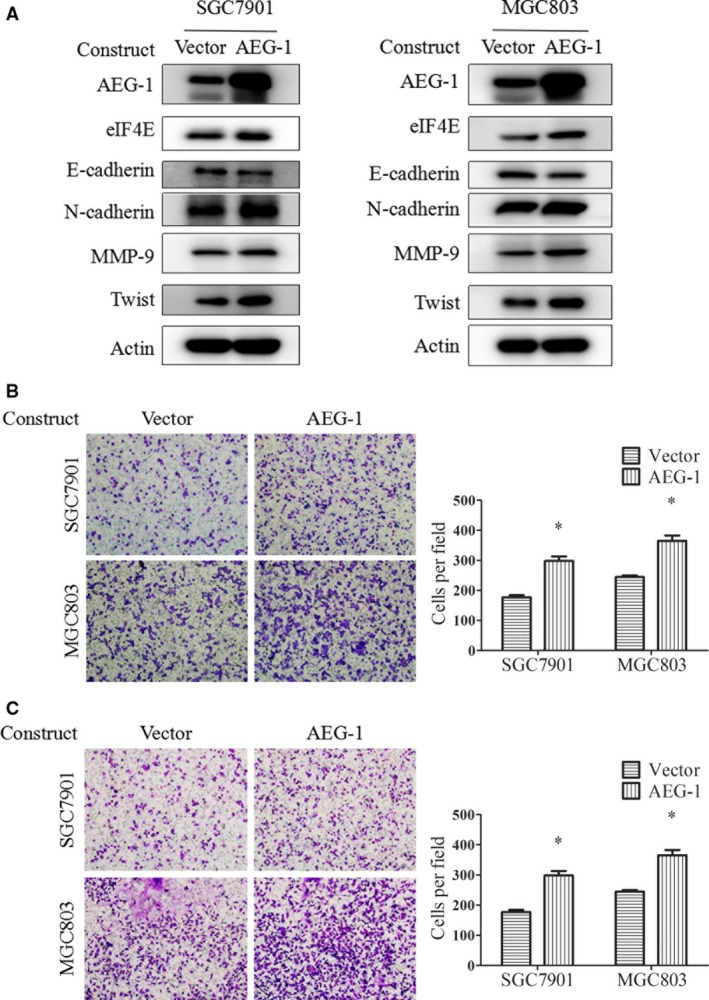
Overexpression of AEG‐1 induced EMT, migration and invasion of gastric cancer cells. **A**, SGC7901 and MGC803 cells were transiently transfected with AEG‐1 constructs or the control vector for 48 hrs and then subjected to Western blot assay. **B** and **C**, SGC7901 and MGC803 cells after transfection of AEG‐1 constructs or control vector for 24 hrs were reseeded to the upper chamber in serum‐free medium and subjected to transwell migration (**B**) and Matrigel invasion (**C**) assays. Magnification, 100×. Columns, means of five fields; bars, S.D. *P < 0.05. The data are representatives of three independent experiments.

To further investigate the role of AEG‐1 in gastric cancer metastasis, we tested the effects of exogenous overexpression of AEG‐1. Transfection of constructs encoding full‐length AEG‐1 resulted in increased AEG‐1 protein levels compared with the control vector in the SGC7901 and MGC803 cells, based on Western blot assays (Fig. 2A). In parallel, E‐cadherin decreased and N‐cadherin increased in the AEG‐1‐overexpression cells. Moreover, AEG‐1 overexpression promoted cell migration and invasion, according to the transwell migration assay (Fig. 2B) and the Matrigel invasion assay (Fig. 2C). Statistical significance was observed after cell quantification. Finally, AEG‐1 overexpression increased eIF4E expression (Fig. 2A). These results suggest that AEG‐1 promotes EMT, migration and invasion, and positively regulates eIF4E expression.

### *Knockdown of eIF4E expression partially abrogated AEG‐1*‐*induced EMT, migration and invasion of gastric cancer cells*

**Figure 3:**
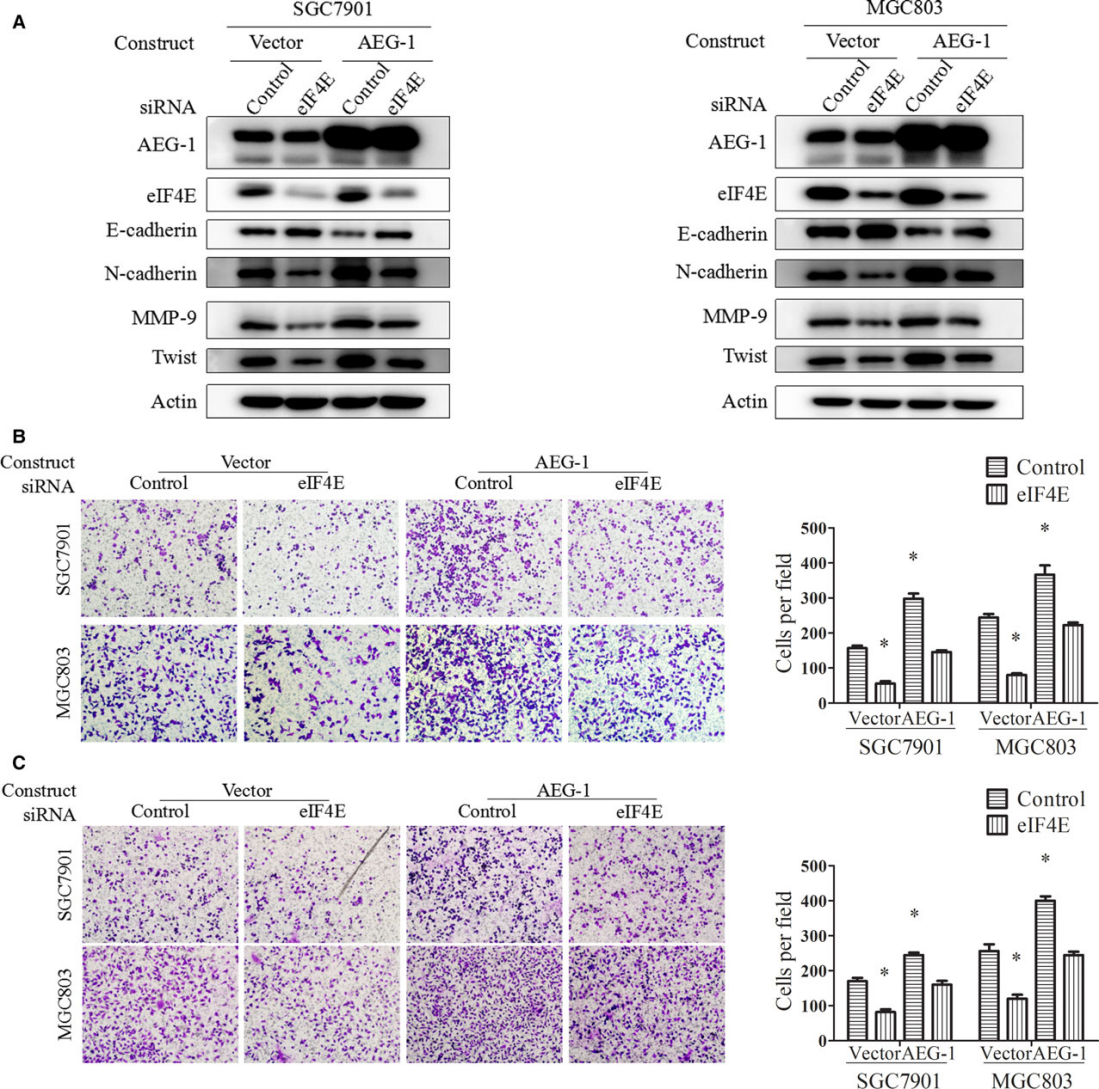
Knockdown of eIF4E partially abrogated AEG‐1‐induced EMT, migration and invasion. SGC7901 and MGC803 cells were transiently transfected with AEG‐1 constructs or the control vector for 24 hrs, then reseeded and transfected with eIF4E siRNA or control siRNA for another 24 hrs. Cells then underwent Western blot (**A**), migration (**B**) and invasion (**C**) assays. Magnification, 100×. Columns, means of five fields; bars, S.D. *P < 0.05. The data are representatives of three independent experiments.

To clarify the role of eIF4E in AEG‐1 signalling, we first determined the effect of AEG‐1 when siRNAs were used to interfere with eIF4E expression. As shown in Figure 3A, AEG‐1 construct transfection increased AEG‐1 protein levels, while eIF4E siRNA transfection significantly decreased eIF4E protein levels in the SGC7901 and MGC803 cells. Moreover, AEG‐1 overexpression‐induced downregulation of E‐cadherin and upregulation of N‐cadherin were greatly abrogated by eIF4E knockdown (compare lane 4 with lane 3 in Fig. 3A). Finally, AEG‐1 overexpression‐induced cell migration (Fig. 3B) and invasion (Fig. 3C) were partially abrogated by eIF4E knockdown. Cell quantification showed statistical significance. These results suggest that eIF4E plays an important role in mediating AEG‐1‐induced EMT, cell migration and invasion.

### *Overexpression of eIF4E promoted cell migration and invasion in AEG‐1*‐*silencing cells*

**Figure 4:**
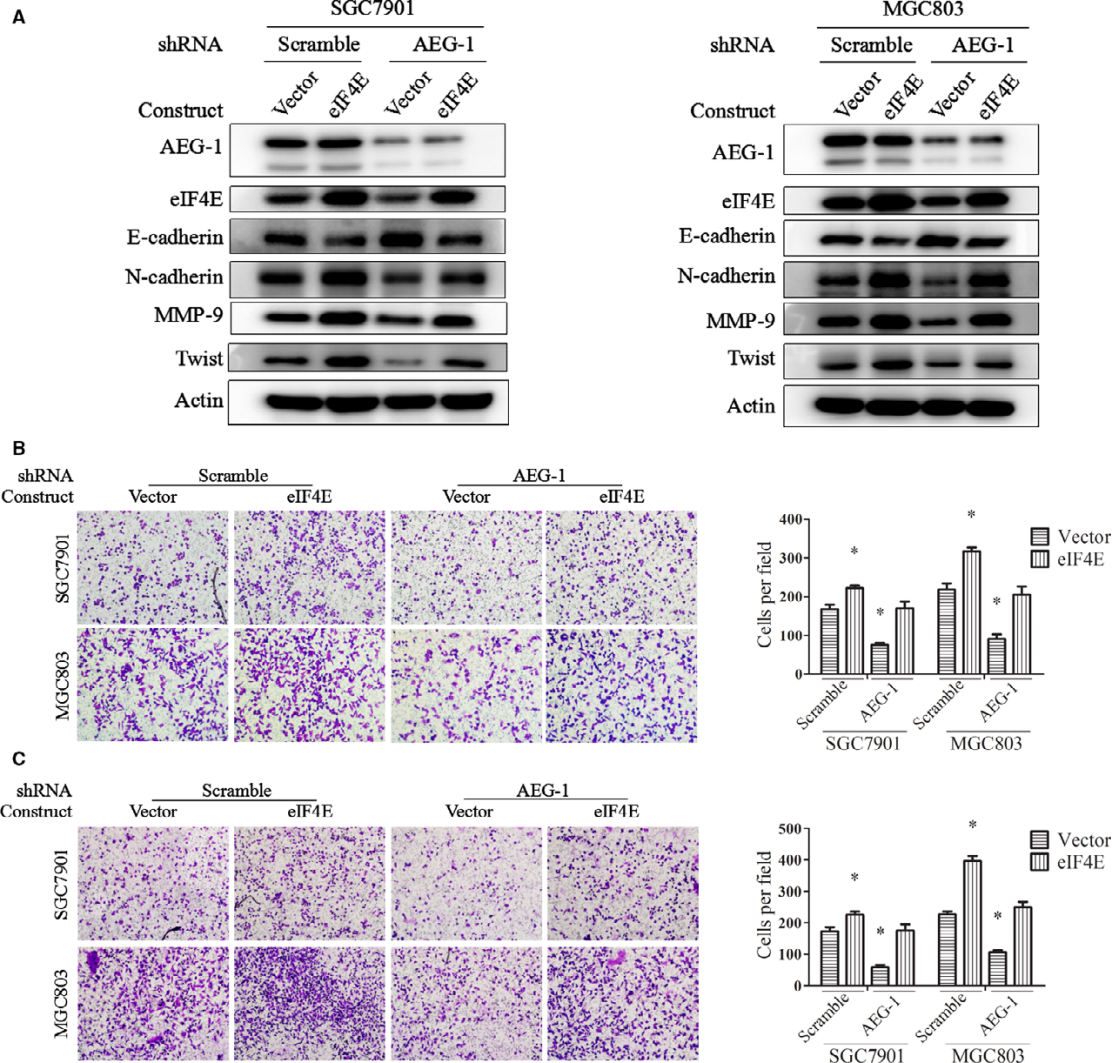
Overexpression of eIF4E partially reversed AEG‐1 silencing‐induced suppression of EMT, migration, and invasion. SGC7901 and MGC803 cells with stable AEG‐1 knockdown (AEG‐1_shRNA) or the control cells (scramble_shRNA) were transiently transfected with eIF4E overexpression constructs or the control vector for 24 hrs and then subjected to Western blot (**A**), migration (**B**) and invasion (**C**) assays. Magnification, 100×. Columns, means of five fields; bars, S.D. *P < 0.05. The data are representatives of three independent experiments.

We also evaluated the effect of eIF4E in SGC7901 and MGC803 cells with stable AEG‐1 knockdown. SGC7901‐AEG‐1_shRNA and MGC803‐AEG‐1_shRNA cells, as well as the control cells, were transiently transfected with constructs inserted with full‐length eIF4E. Western blot assays showed that eIF4E protein levels were significantly increased, suggesting successful overexpression of eIF4E (Fig. 4A). Moreover, elevated E‐cadherin and decreased N‐cadherin protein levels in AEG‐1‐silencing cells were partially reversed by eIF4E overexpression (Fig. 4A). Finally, eIF4E overexpression promoted cell migration (Fig. 4B) and invasion (Fig. 4C) in the control cells and in the AEG‐1‐silencing cells, suggesting that eIF4E overexpression reversed AEG‐1′s effects on EMT, cell migration and invasion.

In addition, to identify the possibility that eIF4E also regulated AEG‐1, we measured AEG‐1 expression by eIF4E knockdown or overexpression. As shown in Figures 3A and 4A, knockdown of eIF4E or exogenous overexpression of eIF4E did not change the protein levels of AEG‐1. These results suggest that eIF4E is located downstream of AEG‐1 and mediates AEG‐1′s function in EMT, migration and invasion.

### EIF4E up‐regulated MMP‐9 and Twist expression to promote EMT, cell migration and invasion

**Figure 5:**
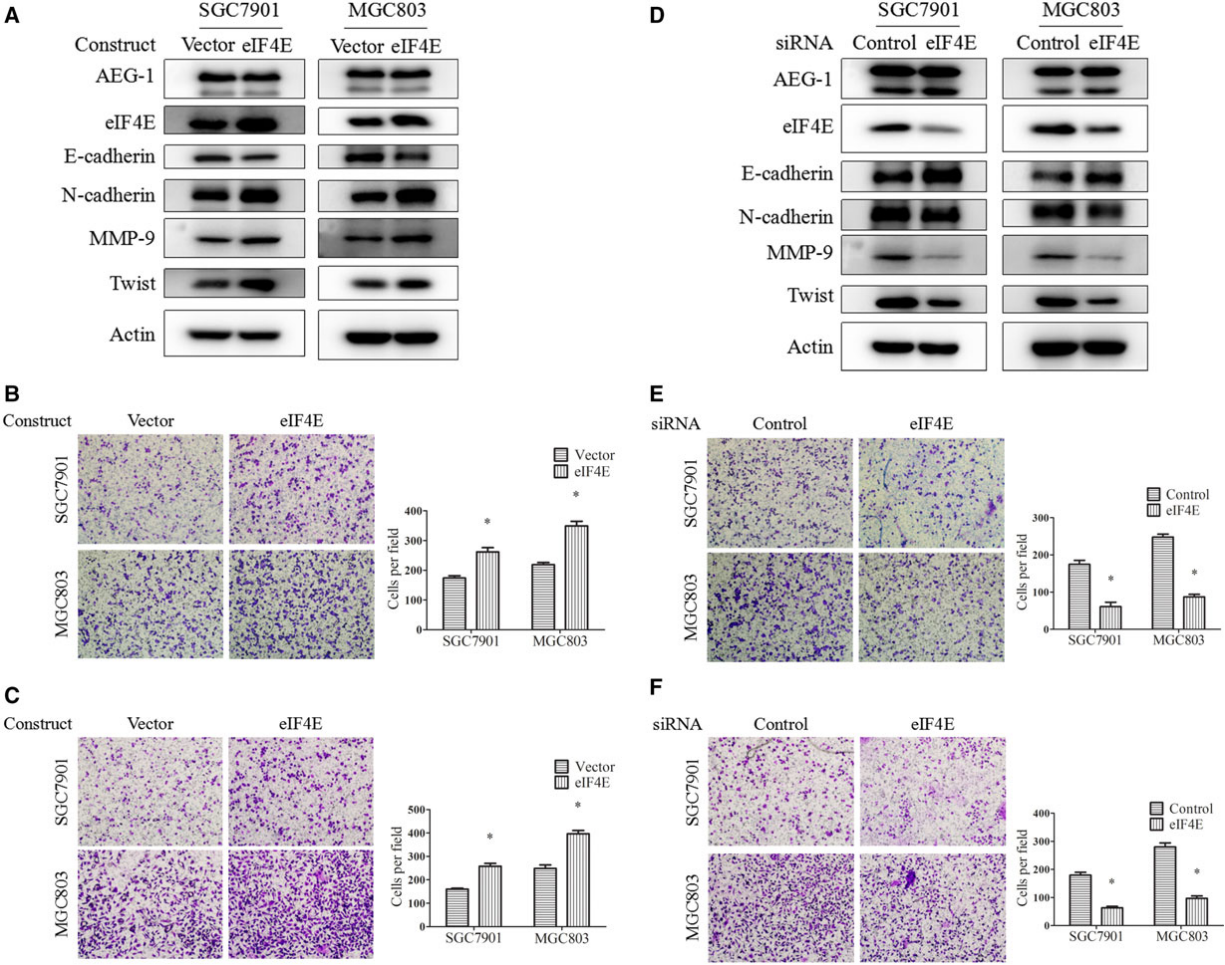
Overexpression or knockdown of eIF4E expression positively regulated EMT, migration and invasion of gastric cancer cells in parallel with regulation of MMP‐9 and Twist expression. **A**,** B** and **C**, SGC7901 and MGC803 cells were transiently transfected with eIF4E overexpression constructs or the control vector for 48 hrs, then subjected to Western blot (**A**), migration (**B**) and invasion (**C**) assays. (**D**,** E** and **F**), these two cell lines were transiently transfected with eIF4E siRNA or control siRNA for 24 hrs and then subjected to Western blot (**D**), migration (**E**) and invasion (**F**) assays. Magnification, 100×. Columns, means of five fields; bars, S.D. *P < 0.05. The data are representatives of three independent experiments.

We have reported that elevated eIF4E protein expression in human gastric cancer tissue is correlated with cancer metastasis 30. However, the mechanism of eIF4E in metastasis has not been fully clarified. In SGC7901 and MGC803 cells, transfection of eIF4E‐overexpression constructs resulted in decreased E‐cadherin and increased N‐cadherin, suggesting induction of EMT (Fig. 5A). Also, eIF4E overexpression promoted cell migration and invasion according to the transwell migration assay (Fig. 5B) and the Matrigel invasion assay (Fig. 5C). In contrast, knockdown of eIF4E expression by siRNAs increased E‐cadherin and decreased N‐cadherin protein levels (Fig. 5D) and suppressed cell migration and invasion (Fig. 5E and F). These results suggest that eIF4E promotes EMT, cell migration and invasion in gastric cancer, consistent with findings in other types of cancers.

For the downstream factors mediating eIF4E's effects, we examined candidates including MMP‐9, MMP‐2 and Snail, which have been proven to be directly regulated by eIF4E in mRNA translation levels, and are also key mediators of cancer metastasis 38, 39. We found that MMP‐9 was up‐regulated by eIF4E overexpression and down‐regulated by eIF4E knockdown (Fig. 5A and D). We also detected its expression in AEG‐1 knockdown or overexpression cells. As shown in Figures 1A and 2A, knockdown or overexpression of AEG‐1 positively regulated MMP‐9 expression. Moreover, the AEG‐1 overexpression‐induced MMP‐9 was abrogated by eIF4E knockdown (Fig. 3A), and silencing of AEG‐1‐reduced MMP‐9 was reversed by eIF4E overexpression (Fig. 4A). However, MMP‐2 and Snail were not regulated in the same pattern as MMP‐9 (data not shown).

Notably, we found that another key EMT molecule, Twist, was positively regulated by AEG‐1 (Figs 1A and 2A) and eIF4E (Fig. 5A and D). Moreover, the up‐ or downregulation of Twist by AEG‐1 could be reversed by eIF4E knockdown or overexpression, respectively, in the same pattern as MMP‐9 (Figs 3A and 4A). These results suggest that eIF4E regulates MMP‐9 and Twist, which mediate the AEG‐1‐induced EMT, migration and invasion of gastric cancer cells.

### Silencing of AEG‐1 expression inhibited gastric cancer metastasis and down‐regulated eIF4E, MMP‐9 and Twist expression in an orthotopic nude mouse model

To further confirm the effect of AEG‐1 on gastric cancer metastasis *in vivo*, an orthotopic nude mouse model was used. As inoculating SGC7901 cells directly to the subserosal layer of the greater gastric curvature results in almost negative lymph node or peritoneal metastasis, we used a model reported by Hu *et al*. 37 Briefly, SGC7901‐AEG‐1_shRNA cells with stable AEG‐1 silencing and the control SGC7901‐scramble_shRNA cells were inoculated subcutaneously into the flank region of nude mice to establish a xenograft mouse model. When the diameter of the xenografts reached 5 mm, tumours were isolated and sheared into 1–2 mm^3^ pieces. These tumour fragments were again inoculated subcutaneously into the xenograft nude mouse model. After five rotations, the xenografts were transplanted into the subserosa of the greater gastric curvature of the mice for 30 days. When the tumour could be felt on the abdomen, the mice were killed and the numbers of lymph node and peritoneal metastases were calculated.

**Figure 6:**
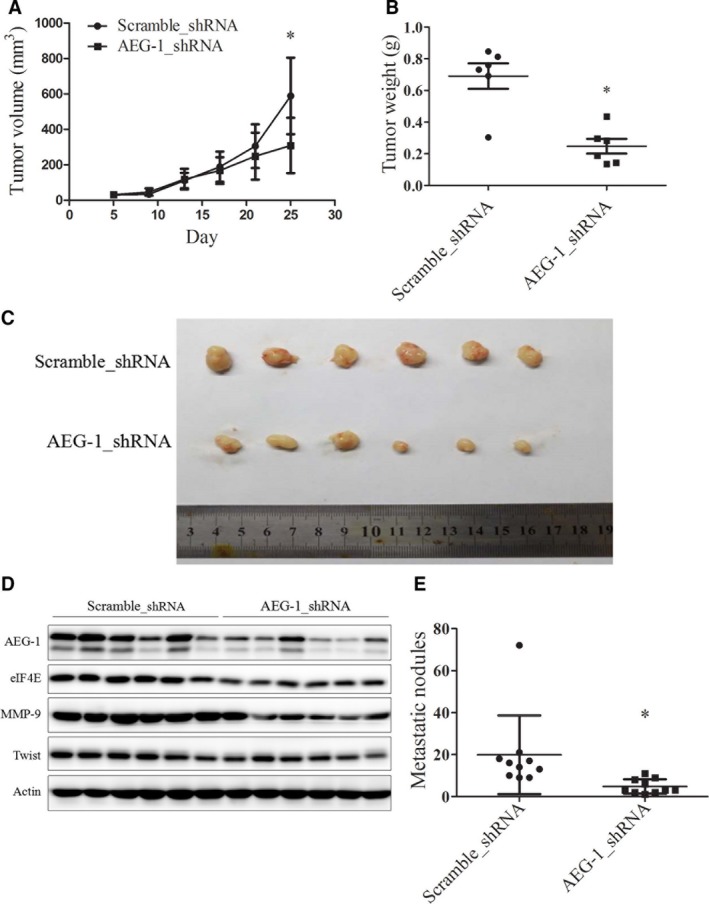
Silencing of AEG‐1 expression suppressed tumour growth and metastasis in xenograft and orthotopic nude mouse models, respectively, in parallel with downregulation of eIF4E, MMP‐9 and Twist protein levels. **A**,** B** and **C**, tumour size (**A**), tumour weight (**B**) and tumour images (**C**) of initial AEG‐1 knockdown (AEG‐1_shRNA) and control tumours (scramble_shRNA) evaluated in the xenograft nude mouse model. Points, means of six tumours; bars, S.D. (**A**). Horizontal lines, means of six tumours; points, tumour weight of each mouse (**B**). **D**, cell lysates of each tumour were prepared and subjected to Western blot assay. **E**, the number of metastases from AEG‐1 knockdown tumours and control tumours were quantified in the orthotopic nude mouse model. Horizontal lines, means of 10 mice; points, metastatic tumour number in each mouse. *P < 0.05.

In the initial xenograft mouse model, tumour size and weight were evaluated. As shown in Fig. 6A, the average tumour size was smaller in the AEG‐1‐silencing group than in the control group, and the difference was statistically significant on day 25. The average tumour weight was also much lower in the AEG‐1‐silencing group than in the control group (0.69 ± 0.20 g *versus* 0.25 ± 0.11 g) (Fig. 6B). Figure 6C shows an image of all of the tumours, demonstrating that the AEG‐1‐knockdown tumours were much smaller than the control tumours. Moreover, levels of the AEG‐1, eIF4E, MMP‐9 and Twist proteins were all down‐regulated in the AEG‐1‐silencing group compared with the control group according to Western blot assays (Fig. 6D). These results suggest that knockdown of AEG‐1 expression inhibits tumour growth and down‐regulates eIF4E, MMP‐9 and Twist signalling *in vivo*. Notably, in the orthotopic nude mouse model, the number of metastatic lymph node and peritoneal tumours was lower in the AEG‐1‐silencing group than in the control group (Fig. 6E). Representative images of each nude mouse with metastatic tumours are provided in Data S2, indicated by black arrows. H&E staining was performed to confirm the metastatic nodules and representative images are shown in Data S2. The results suggest that knockdown of AEG‐1 expression suppresses gastric cancer metastasis *in vivo*.

## Discussion

In this study, we defined the role of AEG‐1 in gastric cancer metastasis. First, we found that elevated expression of AEG‐1 was correlated with gastric cancer metastasis in human specimens on immunostaining. Second, we found that in SGC7901 and MGC803 cells, permanent silencing of AEG‐1 expression inhibited EMT (with increased E‐cadherin and decreased N‐cadherin), migration and invasion. Third, overexpression of AEG‐1 by transient transfection of AEG‐1 constructs promoted the EMT, migration and invasion of gastric cancer cells. Fourth, AEG‐1‐silencing SGC7901 tumours showed decreased size and weight compared to the control tumours in a xenograft nude mouse model. Finally, AEG‐1‐silencing tumours showed fewer lymph node metastases and less peritoneal transplantation. These data suggest that AEG‐1 facilitates gastric cancer metastasis through induction of EMT, migration and invasion.

Elevated AEG‐1 expression has been detected in a variety of tumours, which is thought to be correlated to clinical staging, metastasis and prognosis. We have previously reported that AEG‐1 was overexpressed in gastric dysplasia and cancer among 87 cases of gastric adenocarcinoma, 60 cases of dysplasia and 47 cases of normal gastric mucosal tissues; however, we did not analyse its correlation with metastasis 16. In the present study, we re‐read these sections with AEG‐1 immunostaining and performed statistical analyses. The results showed that AEG‐1 expression was positively correlated with gastric cancer metastasis. Recently, Dong *et al*. determined that high AEG‐1 expression was associated with clinical staging, metastasis, and unfavourable prognosis in gastric carcinoma in 119 specimens of cancerous tissue and compared to corresponding distant normal gastric mucosal tissues, suggesting AEG‐1 as a novel predictor for metastasis and prognosis in gastric cancer 17. Although protein expression studies on human samples have been compelling, research on AEG‐1 overexpression and silencing, as well as a knockout mouse model to clarify the effects and mechanisms of AEG‐1 in gastric cancer, has been limited and is urgently needed. We previously reported that knockdown of AEG‐1 expression using siRNA inhibited the growth of gastric cancer cells through downregulation of the cell‐cycle‐checkpoint protein cyclin D1 16. Xu *et al*. reported that AEG‐1 was involved in the Wnt/β–catenin signalling pathway for the growth and apoptosis of gastric cancer cells 40. Li *et al*. found that AEG‐1 promoted progression of gastric cancer through LPS/TLR4/p65‐NFκB‐mediated inflammation 41. Li *et al*. reported that AEG‐1 up‐regulated VEGF and HIF1α expression, promoting angiogenesis in gastric cancer 42. The mechanism of AEG‐1 in promoting cancer metastasis currently remains elusive.

In the process of EMT, cells lose the epithelial marker E‐cadherin and gain some mesenchymal markers, such as N‐cadherin, vimentin, Snail and Twist; this subsequently increases the cell's capacity for migration and invasion, separate from its original location. EMT is the first and the key step in cancer metastasis. The promotion of metastasis by AEG‐1‐induced EMT has been demonstrated in a variety of malignancies. Combined detection of AEG‐1 and EMT biomarkers in the same species of human tissues, and comparisons of their expression in metastatic tissues with non‐metastatic tissues, has been done on patient samples to reveal their relationship 43. Negative correlations between AEG‐1 and E‐cadherin, or positive correlations between AEG‐1 and vimentin, have been identified in hepatocellular carcinoma 44, lung cancer 45, laryngeal squamous cell carcinoma 46, squamous cell carcinoma of the head and neck 47 and osteosarcoma 48. Other studies have confirmed that overexpression or downregulation of AEG‐1 expression regulates EMT, migration or invasion *in vitro* in liver cancer cells 43, head and neck squamous cells 47, cervical cancer cells 49, and lung cancer cells 35, and in animal breast cancer models 50. However, similar studies on gastric cancer are lacking. The present study demonstrated that manipulating AEG‐1 expression regulated EMT markers and the cell's migration and invasion capabilities.

Candidates for the downstream signals of AEG‐1 include PI3K/Akt, MAPK/ERK, NF‐κB, Wnt, c‐Myc and cyclin D1 20. We have previously confirmed that cyclin D1 is regulated by AEG‐1 in gastric cancer 16. However, the detailed mechanism is still unknown. EIF4E activation is the rate‐limiting step in translation initiation and facilitates the metastasis of numerous types of cancer. We have previously reported that it is overexpressed in gastric cancer, promotes cell growth and is inhibited by perifosine, an Akt inhibitor, in a pattern similar to that of AEG‐1 30. In this study, we found that overexpression of AEG‐1 increased eIF4E expression, and knockdown of eIF4E levels partially abrogated AEG‐1‐induced EMT, migration and invasion. In addition, silencing of AEG‐1 decreased eIF4E expression, while overexpression of eIF4E partially rescued AEG‐1′s effects. Moreover, overexpression or knockdown of eIF4E did not affect AEG‐1 protein levels. These results suggest that downregulation of eIF4E plays an important role in AEG‐1‐induced EMT, migration, and invasion.

In this study, although eIF4E mRNA and protein expression were positively regulated by AEG‐1, the mechanism was more complicated than expected. C‐Myc is the only transcriptional factor proven to activate the eIF4E promoter by binding to the E‐box (CACGTG) of eIF4E, leading us to suspect that AEG‐1 may up‐regulate eIF4E through c‐Myc 28. However, our previous work revealed that c‐Myc was not located downstream of AEG‐1 in gastric cancer 16. We then predicted the transcription factors in the eIF4E promoter (1.0 kb length upstream of the starting code) using Alibaba 2.1 software. The results showed that transcription factors able to bind directly to the eIF4E promoter included NFκB, C/EBPα and β, AP‐1, SP‐1, RXRβ and Oct‐1. Previous studies showed that by activating PI3K/Akt signalling, AEG‐1 activated the transcription factors FoxO1, FoxO3a, NFκB and AP‐1 32, 51, 52. Moreover, AEG‐1 directly interacted with p65‐NFκB, SND1 and PLZF to regulate gene transcription 50, 53, 54. Furthermore, by activating the ERK signalling pathway, AEG‐1 activated RXR‐mediated transcription 55. In addition, in the context of epigenetic development, an examination of the regulation by the histones or promoter DNA modifications of the eIF4E promoter, such as acetylation or methylation, would be interesting. Clarification of the mechanism of AEG‐1‐regulated eIF4E is worthy of further investigation.

Downstream targets of eIF4E that mediate its pro‐metastatic functions include MMP‐9, MMP‐2, Snail, c‐Myc and VEGF 39. As a key component of the eukaryotic translation initiation factor complex, which is bound directly to the long 5′‐UTR region of these molecules' mRNA, eIF4E promotes their translation 38. However, the signals involved in gastric cancer are not quite clear. In this study, we found that MMP‐9 and Twist protein levels were positively regulated by eIF4E and AEG‐1. Furthermore, manipulating eIF4E expression reversed AEG‐1‐regulated protein levels of these two molecules, suggesting that they were located downstream of eIF4E, mediating AEG‐1′s effects. Although Snail was reported to be a downstream target of AEG‐1 or eIF4E in other types of cancers, it was not the key molecule involved in the AEG‐1/eIF4E pathway in gastric cancer metastasis in this study, as it did not changed in the same pattern as MMP‐9 and Twist did. Twist has been reported to inhibit E‐cadherin transcription and to facilitate EMT; however, its regulation by eIF4E has not been reported 56. As the 5′‐UTR region of Twist mRNA is long, it is possible that eIF4E regulates its mRNA translation, but these mechanisms require further investigation. This study's findings suggest that in gastric cancer, AEG‐1 promotes EMT *via* eIF4E‐regulated MMP‐9 and Twist.

We also conducted an orthotopic nude mouse model to confirm our findings *in vivo*. First, SGC7901 cells were inoculated subcutaneously to establish a xenograft mouse model. After xenografts were grown in the mice for five generations, they were transplanted into the subserosa of the greater gastric curvature and observed for metastasis. This is an orthotopic mouse model that mimics gastric cancer metastasis, including lymph node and peritoneal transplantation metastases 37, 49. Using the AEG‐1 stable knockdown cell lines in this model, we revealed that silencing of AEG‐1 expression not only inhibited tumour growth in parallel with decreased expression of eIF4E, MMP‐9 and Twist, but it also inhibited the lymph node and peritoneal metastasis of gastric cancer.

In summary, the present study demonstrates that AEG‐1 promotes EMT and metastasis of gastric cancer through upregulation of eIF4E‐mediated expression of MMP‐9 and Twist. Our findings clarify a new mechanism of AEG‐1 in gastric cancer metastasis and provide new targets for gastric cancer therapies.

## Conflicts of interest

None.

## Supporting information


**Data S1** Stable cell lines with AEG‐1 knockdown suppressed migration and invasion of gastric cancer cells.
**Data S2** Images and representative H&E staining of metastatic tumors in orthotopic nude mice.
