# Supplementary material for: AEG‐1 induces gastric cancer metastasis by upregulation of eIF4E expression
Source: J Cell Mol Med. 2017 Jun 29;21(12):3481–93. doi: 10.1111/jcmm.13258 (PMC5706588; doi:10.1111/jcmm.13258)
Supplement: Supplementary file 1 — Data S1 Stable cell lines with AEG‐1 knockdown suppressed migration and invasion of gastric cancer cells. Data S2 Images and representative H&E staining of metastatic tumors in orthotopic nude mice. [file JCMM-21-3481-s001.pdf]

Supplementary Data 1

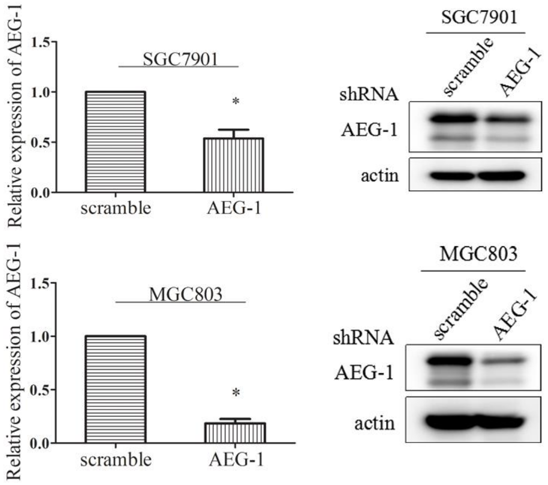

Supplementary Data 1. Stable cell lines with AEG-1 knockdown suppressed migration and invasion of gastric cancer cells. The mRNA and protein expression of AEG-1 in stable AEG-1 knockdown (AEG-1_shRNA) SGC7901 and MGC803 cells or their control cells (scamble_shRNA). Cells were cultured for 24h, and subjected to quantitative reverse transcriptional PCR and western blot assay. Columns, means of three replicates; bars, SD. *, $P<0.05$. The data are representatives of three independent experiments.

Supplementary Data 2.

Images and representative H&E staining of metastatic tumors in orthotopic nude mice

Mouse number	Representative images	Representative H&E staining (100×)
		Number of metastatic nodules
Scramble_shRNA - mouse 1	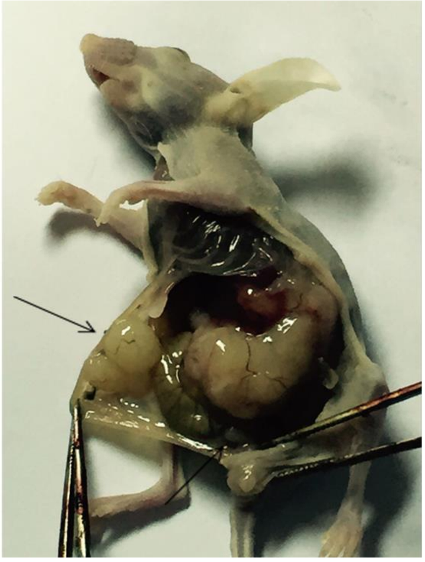	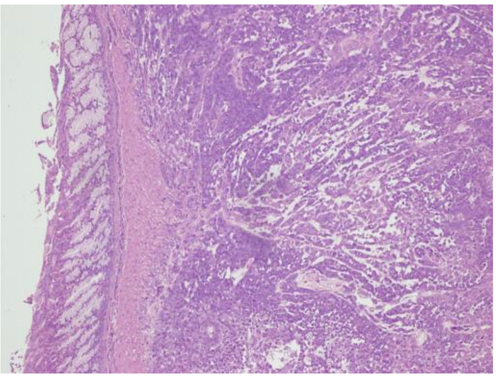
		15
Scramble_shRNA - mouse 2	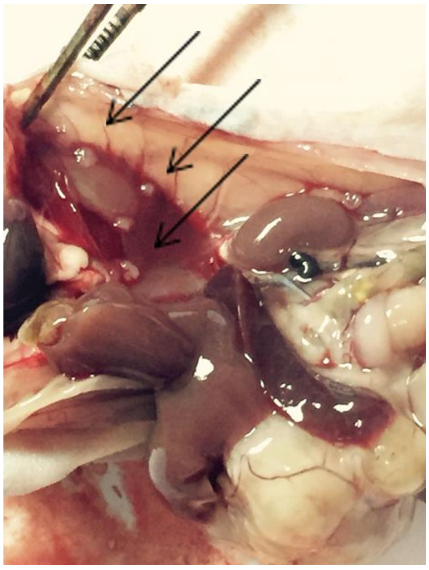	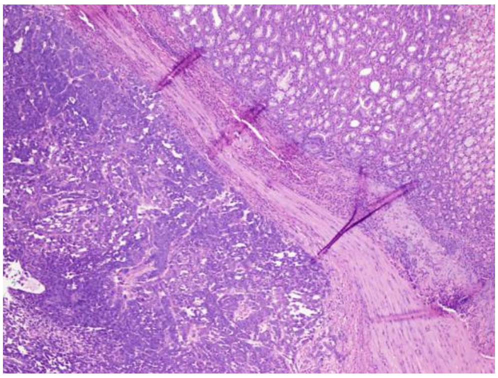
		18

Scramble_shRNA - mouse 3	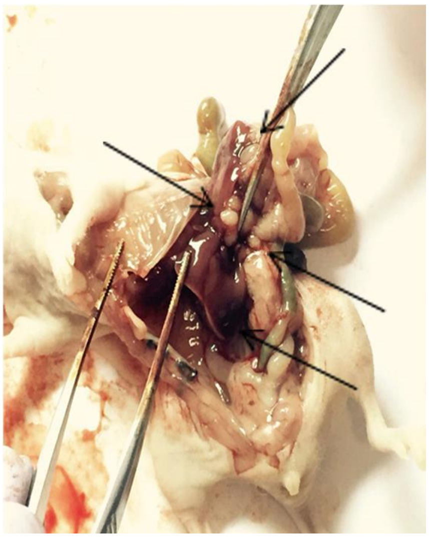	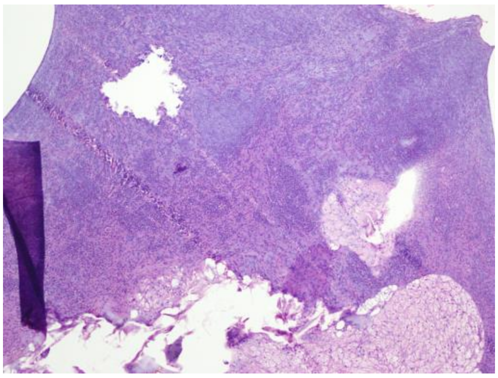<br>21
Scramble_shRNA - mouse 4	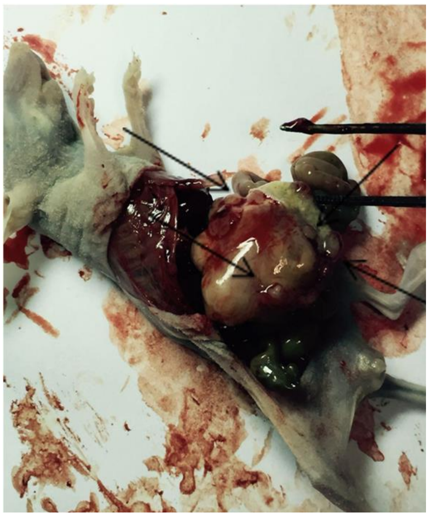	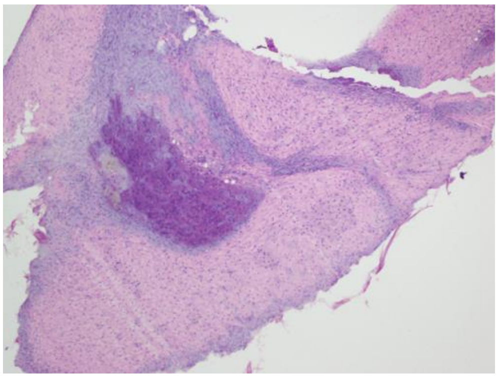<br>13
Scramble_shRNA - mouse 5	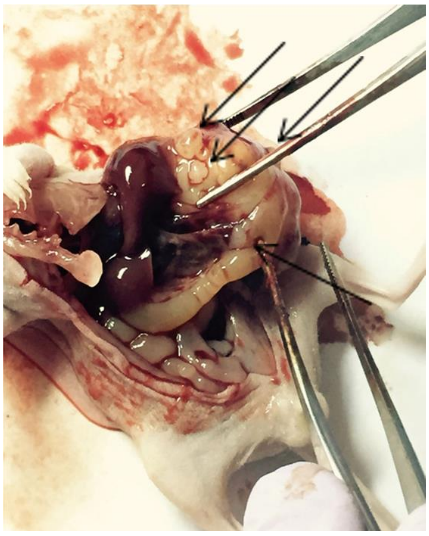	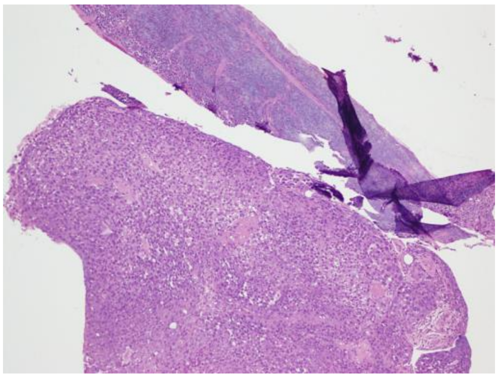<br>16

Scramble_shRNA - mouse 6	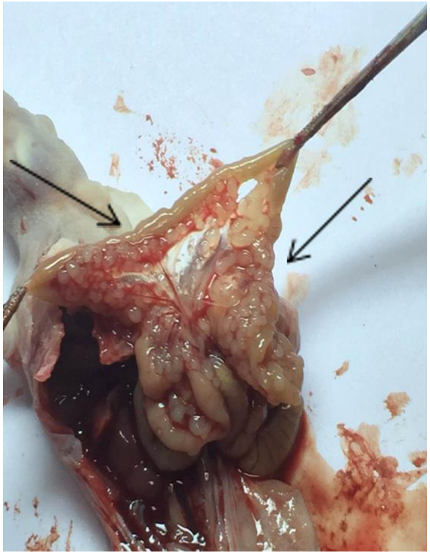	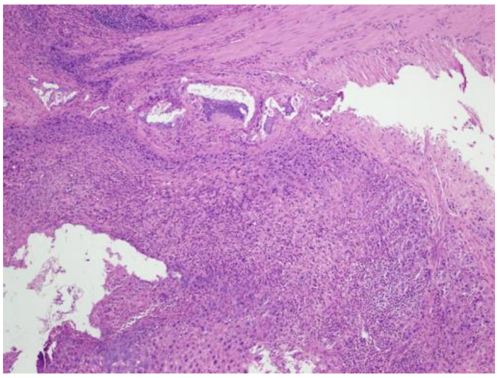<br>72
Scramble_shRNA - mouse 7	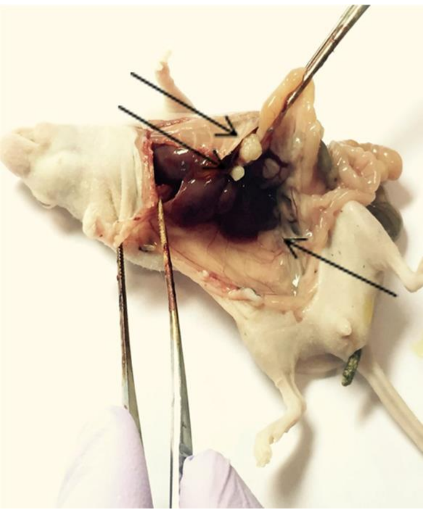	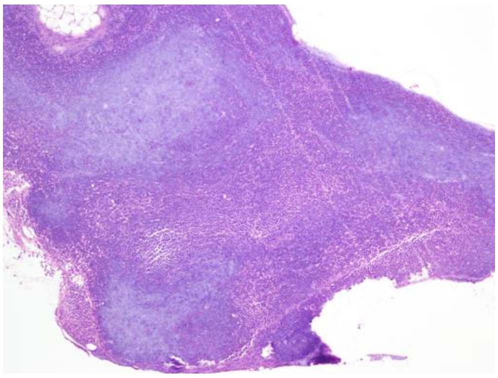<br>9
Scramble_shRNA - mouse 8	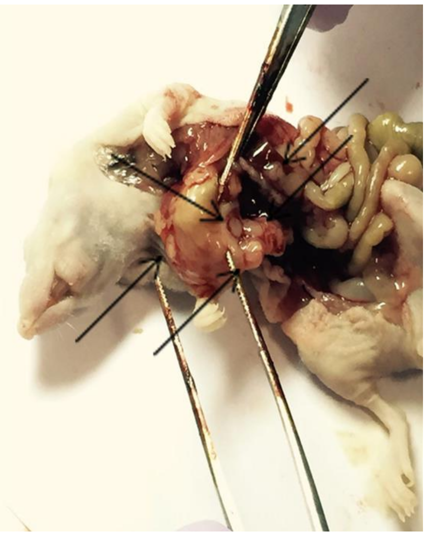	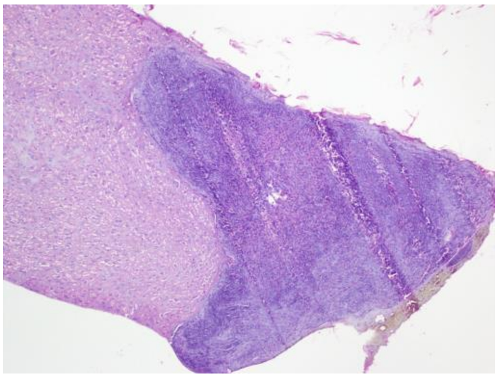<br>9

Scramble_shRNA - mouse 9	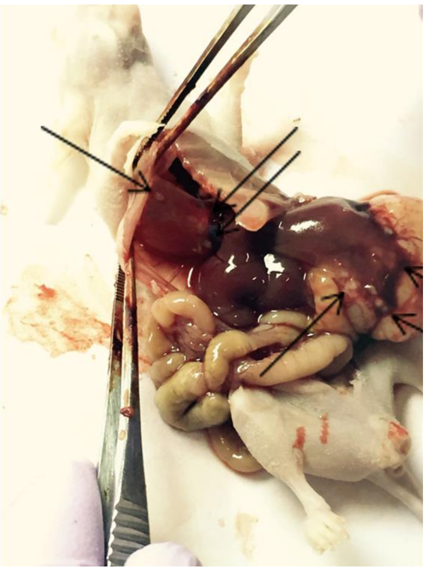	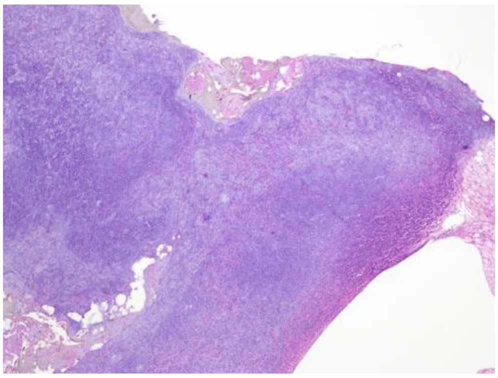<br>17
Scramble_shRNA - mouse 10	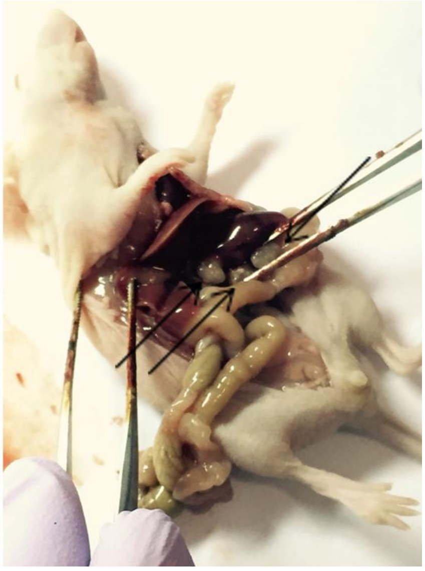	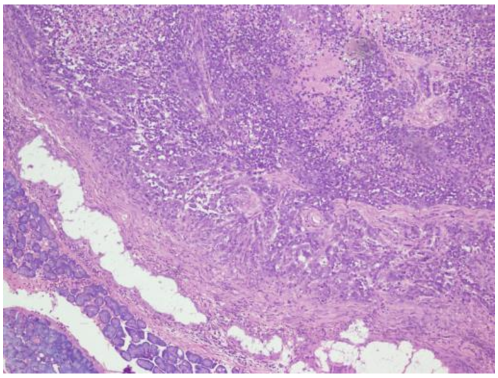<br>11
AEG-1_shRNA - mouse 1	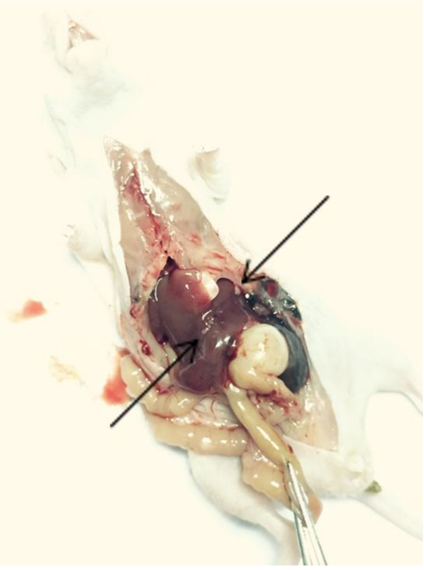	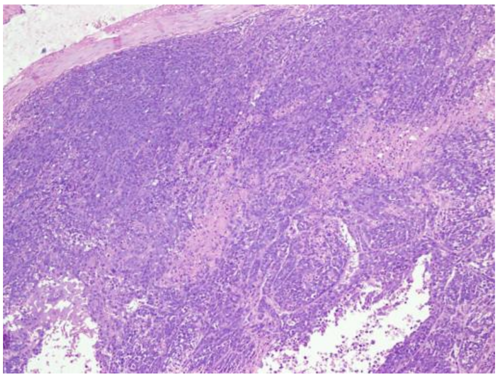<br>11

AEG-1_shRNA - mouse 2	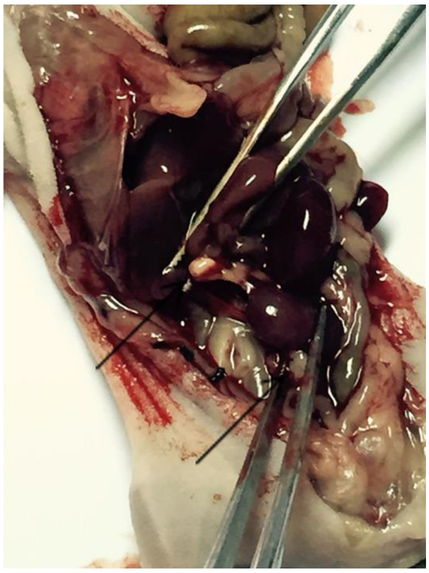	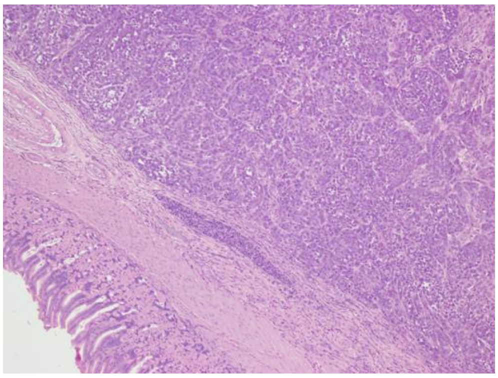<br>3
AEG-1_shRNA - mouse 3	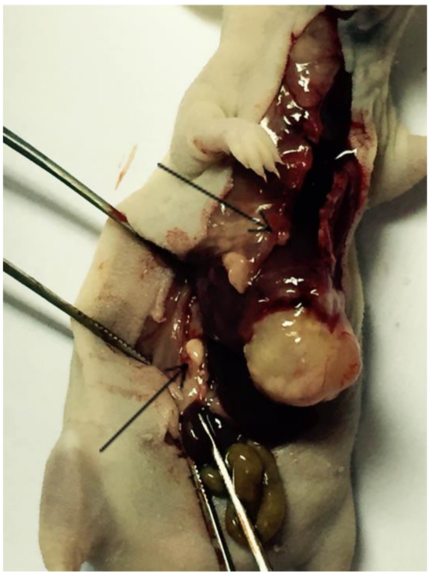	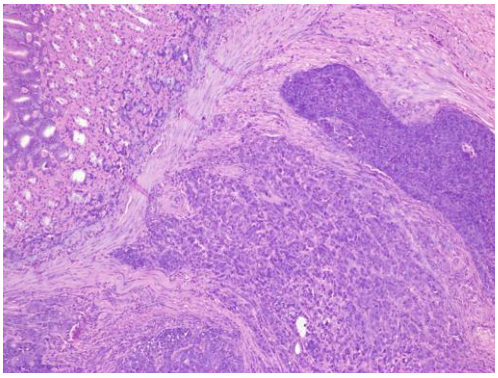<br>2
AEG-1_shRNA - mouse 4	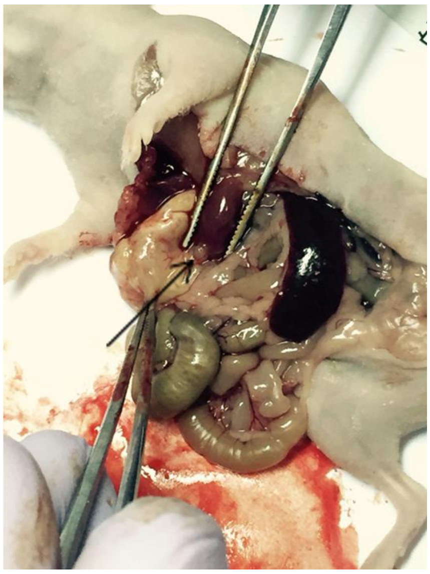	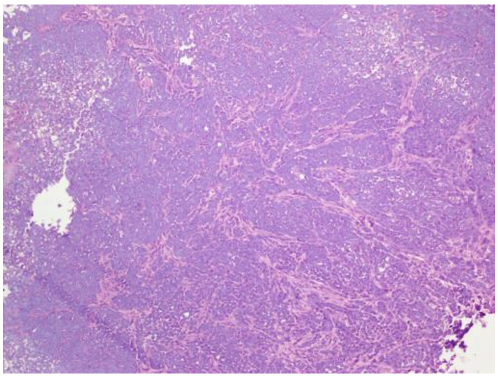<br>8

AEG-1_shRNA - mouse 5	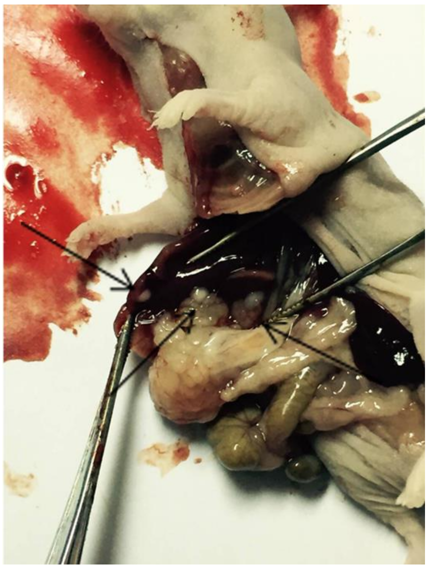	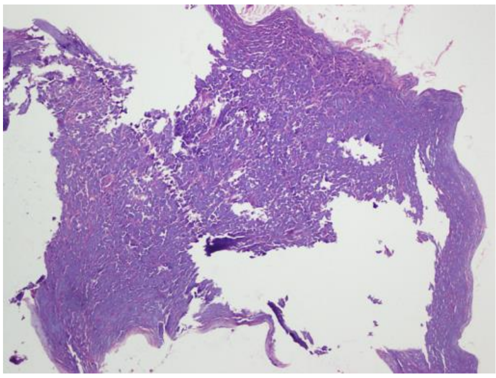<br>9
AEG-1_shRNA - mouse 6	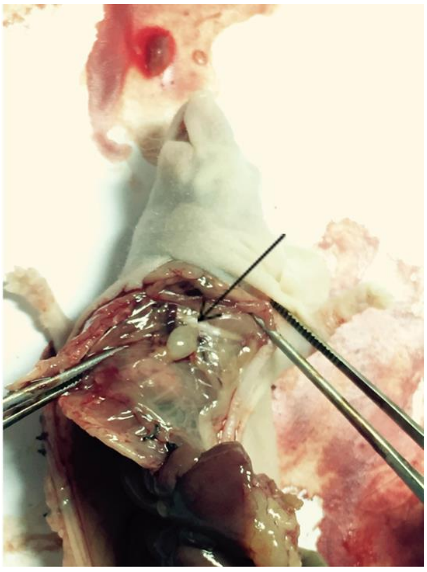	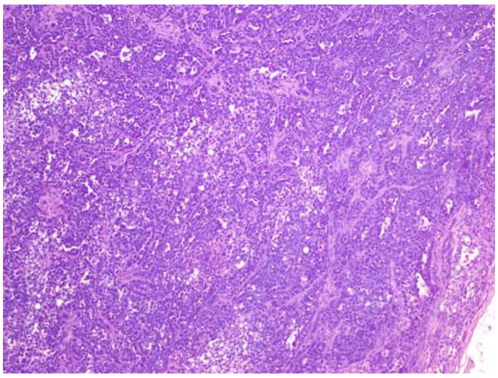<br>3
AEG-1_shRNA - mouse 7	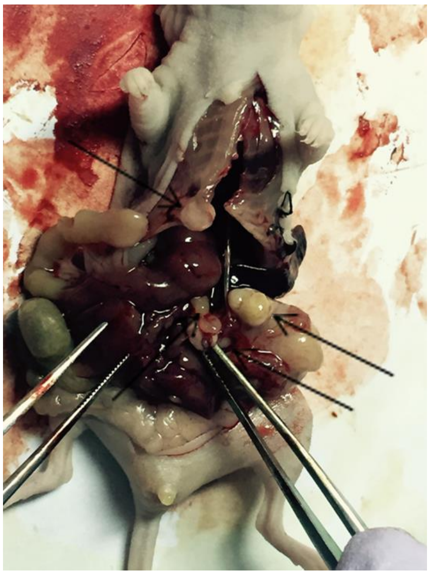	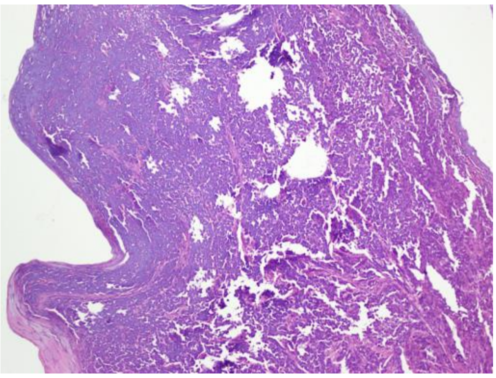<br>6

AEG-1_shRNA - mouse 8	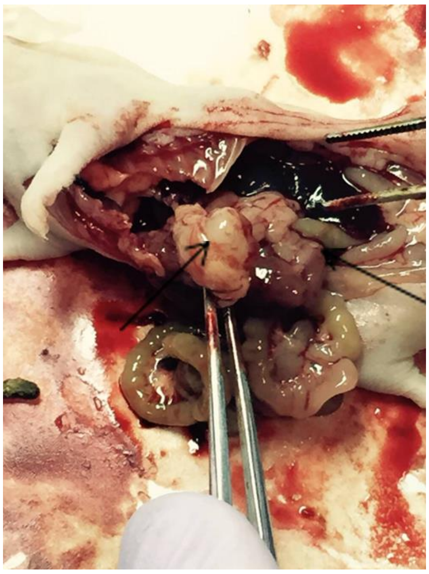	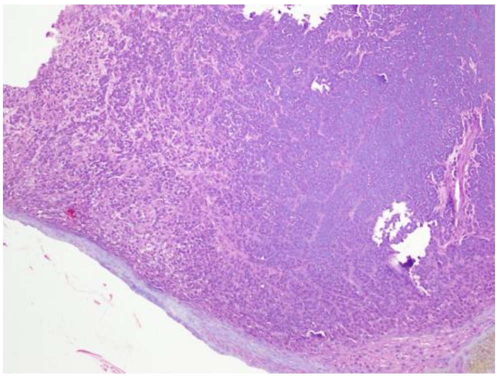	2
AEG-1_shRNA - mouse 9	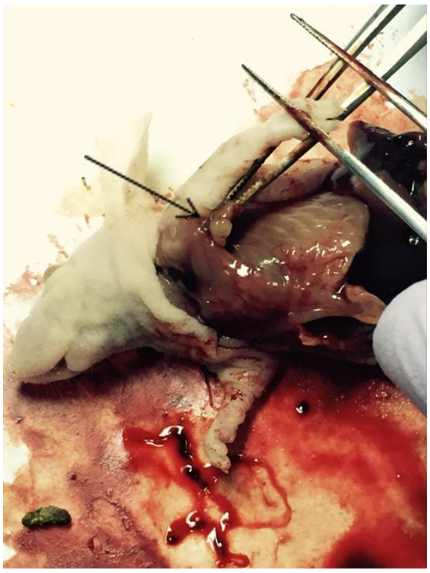	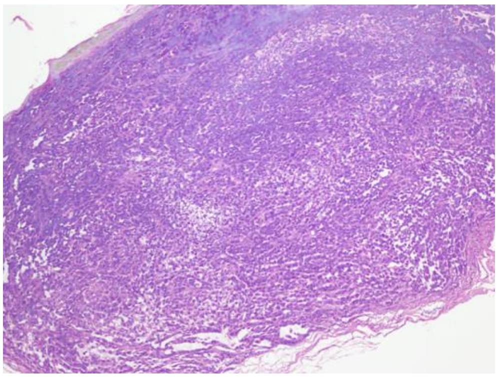	2
AEG-1_shRNA - mouse 10	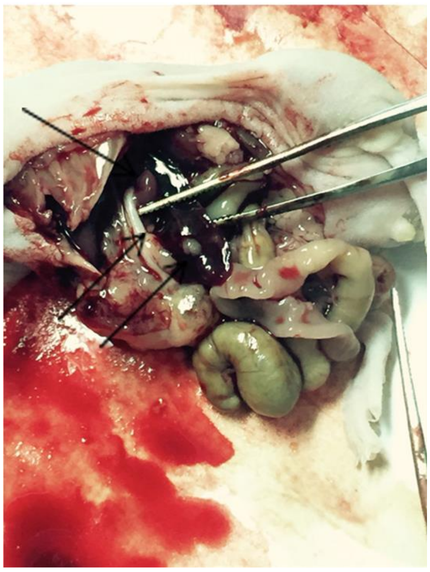	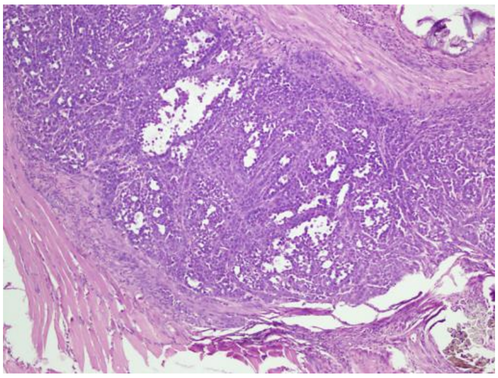	4
